# Survival models with preclustered gene groups as covariates

**DOI:** 10.1186/1471-2105-12-478

**Published:** 2011-12-16

**Authors:** Kai Kammers, Michel Lang, Jan G Hengstler, Marcus Schmidt, Jörg Rahnenführer

**Affiliations:** 1 Department of Statistics, TU Dortmund University, Dortmund, Germany; 2 Leibniz Research Centre for Working Environment and Human Factors (IfADo), TU Dortmund University, Dortmund, Germany; 3 Department of Obstetrics and Gynecology, Mainz University, Medical School, Mainz, Germany

## Abstract

**Background:**

An important application of high dimensional gene expression measurements is the risk prediction and the interpretation of the variables in the resulting survival models. A major problem in this context is the typically large number of genes compared to the number of observations (individuals). Feature selection procedures can generate predictive models with high prediction accuracy and at the same time low model complexity. However, interpretability of the resulting models is still limited due to little knowledge on many of the remaining selected genes. Thus, we summarize genes as gene groups defined by the hierarchically structured Gene Ontology (GO) and include these gene groups as covariates in the hazard regression models. Since expression profiles within GO groups are often heterogeneous, we present a new method to obtain subgroups with coherent patterns. We apply preclustering to genes within GO groups according to the correlation of their gene expression measurements.

**Results:**

We compare Cox models for modeling disease free survival times of breast cancer patients. Besides classical clinical covariates we consider genes, GO groups and preclustered GO groups as additional genomic covariates. Survival models with preclustered gene groups as covariates have similar prediction accuracy as models built only with single genes or GO groups.

**Conclusions:**

The preclustering information enables a more detailed analysis of the biological meaning of covariates selected in the final models. Compared to models built only with single genes there is additional functional information contained in the GO annotation, and compared to models using GO groups as covariates the preclustering yields coherent representative gene expression profiles.

## Background

We present prediction models for survival times built from high dimensional gene expression data. The challenge is to construct models that are complex enough to have high prediction accuracy but that at the same time are simple enough to allow biological interpretation. Univariate approaches use single genes as covariates in survival time models, whereas multivariate models perform dimension reduction through gene selection (see, e.g., [1]). In addition, the combination of clinical data and gene expression data is a hot topic of research [2,3] and is included in our model building procedure. Analysis of the prognostic index [4] and the Brier Score [5,6] can be used to assess the predictive performance of the models.

Here, we present models with higher interpretability by combining genes to gene groups (e.g. biological processes) and then using these groups as covariates in the survival models. The hierarchically ordered 'GO groups' (Gene Ontology) are particularly suitable [7]. The Gene Ontology (GO) project provides structured, controlled vocabularies and classifications according to molecular and cellular biology. The current ontologies of the GO project are *biological process*, *molecular function*, and *cellular component*. These three areas are considered rather independent of each other and we make use of the *biological process *ontology.

A problem when relating gene groups with gene expression profiles is that the genes in each gene group may have different expression profiles: interesting subgroups may not be detected due to heterogeneous or anti-correlated expression profiles within one gene group. We propose to cluster the expression profiles of genes in every gene group and preselect relevant clusters (preclustering).

For statistical analysis, the Cox regression model [8] is a well-known method for modeling censored survival data. It can be used for identifying covariates that are significantly correlated with survival times. Due to the high-dimensional nature of microarray data we cannot obtain the parameter estimates directly with the Cox log partial likelihood approach. However, we can combine the Cox model with selection and shrinkage procedures and compare the prediction performance of the obtained models. Based on these models statistical selection procedures are applied. Univariate selection and forward selection have been shown to have problematic performance in highdimensional settings. Therefore we do not show their results in this work. We focus on presenting the results for ridge regression [9] and lasso regression [10,11] as shrinkage methods. Note that lasso regression is a variable selection method as well.

In order to integrate the clinical information and microarray data in survival models properly, it is a common approach to handle the clinical covariates as unpenalized mandatory variables [3,12]. In addition to the genomic information, clinical covariates like age, tumor size and tumor stage may be important predictors for survival times of patients. These approaches show that the combination of genomic and clinical information may also improve predictions.

Our aim is the combination of methods for survival prediction with biological *a priori *knowledge. On real gene expression data sets we evaluate the potential of including preclustered gene groups as covariates in survival models. Models built with gene groups alone have equal or decreased prediction accuracy since many genes are not yet annotated to their corresponding functions. However, we will show that after adding the preclustering information to the gene groups the resulting models have improved interpretability while prediction performance remains stable.

In the next chapter we introduce the methods for analyzing survival data, for preclustering genes, for model selection, and for evaluating the prediction accuracy of the resulting survival models. Then we present and discuss results on two real gene expression data sets.

## Methods

We first present the notation, the Cox model and how the covariates are defined that are used in the Cox models - especially the preclustering Algorithm is presented. Then we describe the log partial likelihood concept derived for the Cox model and introduce model selection/shrinkage methods. Since most methods for dimension reduction or shrinkage require the selection of a tuning parameter that determines the amount of shrinkage, finally, we describe how to choose the tuning parameter for each method.

### Cox model

In the following, we assume that we have a sample size of *n *patients, and a (possibly right-censored) survival time for the response. In order to cope with censored survival times data we use the Cox model, also known as proportional hazards regression model [8]. Cox suggested that the risk of an event (e.g. cancer recurrence, death or any date of interest) at time *t *for a patient with given covariate vector *x *= (*x*_1_,..., *x_p_*) is modeled as

(1) $$h\left(t|x\right)=h_{0}\left(t\right)\exp\left(\beta^{\prime }x\right),$$

where *h*_0_(·) is an arbitrary baseline hazard function and *β *= (*β*_1_,..., *β_p_*) a vector of regression coefficients. In the classical setting with *n > p*, the regression coefficients are estimated by maximizing the log partial likelihood

(2) $$l\left(\beta \right)=\overset{n}{\underset{i=1}{\sum }}\delta_{i}\left[\beta^{\prime }x_{i}-\log\left(\underset{j\in R\left(t_{i}\right)}{\sum }\exp\left(\beta^{\prime }x_{j}\right)\right)\right].$$

For patient *i*, this expression contains the possibly censored failure time *t_i_*, the (non-censoring-)indicator *δ_i _*(equal to 1 if *t_i _*is a true survival time and to 0 if it is censored) and the vector of gene (or summarized gene group) expression values *x_i_*.

Further, *R*(*t_i_*) is the risk set at time *t_i_*; this is the set of all patients who have not yet failed nor been censored. The value of *β*′*x_i _*is called *prognostic index *or *risk score *of patient *i*.

### Definition of covariates

In the following, we assume that the data consists of two different categories of covariates

clinical covariates *Z *= (*Z*_1 _, *..*., *Z_q_*): e.g. tumor size, tumor grade, age

genomic covariates *X *= (*X*_1_, *..*., *X_p_*): gene expression values of single genes or combined gene expression values for gene groups.

For a detailed analysis we consider three different types of Cox models. We start with the simple model using only the clinical covariates

(3) $$h\left(t|Z\right)=h_{0}\left(t\right)\exp\left(\gamma^{\prime }Z\right).$$

The second model consists of *p *genomic covariates *X *= (*X*_1_,..., *X_p_*). In our genetic regression models we use single genes, gene groups as well as preclustered gene groups as covariates. A gene group must be appropriately summarized in order to obtain one representative value for each individual (patient). We summarize the gene expression measurements from all genes belonging to one GO group or cluster via the first principle component of all genes that belong to this gene group. In the following we will consider three types of genomic models:

genes

groups

preclustered GO groups.

In the last step, we combine the genomic models with the clinical model, which can be written as

(4) $$h\left(t|X,\;Z\right)=h_{0}\left(t\right)\exp\;\left(\beta^{\prime }X+\gamma^{\prime }Z\right)$$

Due to the small number of clinical covariates, the shrinkage and dimension reduction procedures will only be applied to the genomic covariates.

### Preclustering with PAM

In order to find *K *homogeneous subgroups of genes within one GO group containing *N *genes, we use the partitioning around medoids (PAM) cluster analysis (cf. [13]). The PAM procedure is based on the search for *K *representative genes, the medoids, among the *N *genes to be clustered. To achieve the goal of finding *K *medoids that minimize the sum of dissimilarities of the genes to their closest medoid

(5) $$\overset{N}{\underset{i=1}{\sum }}\underset{j=1,...,K}{\min}d\left(i,\;j\right),$$

where *d*(*i, j*) is the dissimilarity of the *i*th and *j*th gene, the two following steps are carried out iteratively until convergence, starting with *K *sequentially selected genes as initial solution:

Build: Select sequentially *K *initial clusters and assign each gene to its closest medoid.

Swap: Minimize the objective function (5) by switching medoids with other genes of the same cluster.

To find correlated subgroups, the dissimilarity

$$d\left(i,j\right)=1-\text{Cor}\left(x_{i},\;x_{j}\right)$$

of the *i*th and *j*th gene with the gene expressions *x_i _*and *x_j _*is based on their Pearson correlation. This yields small dissimilarities between positively correlated genes and large values for negatively correlated genes, respectively.

The number of clusters *K *for the PAM algorithm has to be chosen in advance. To find tight clusters of highly correlated genes, [14] suggest using the Intra Cluster Correlation:

$$\text{ICC}=\frac{2}{n\left(n-1\right)}\underset{i}{\sum }C_{i}.$$

Here, the values *C_i _*are the elements of the lower triangle of the correlation matrix of the *N_j _*genes within a single cluster. The maximum mean ICC among the *K *= 2,..., *N - *1 possible cluster configurations corresponds to the optimal number of clusters within one GO group.

### Methods for dimension reduction

For comparing our results to those being published in the literature, we make use of the following two most established and successful shrinkage procedures: *L*_1 _(lasso) and *L*_2 _(ridge) penalized regression. Univariate and forward stepwise selection do not produce satisfactory results for our high dimensional settings. We have compared these two methods in our analysis, and in agreement with previous results from Boevelstad et al. [4,12] prediction performance was always worse (data not shown). We present the methods for the model containing clinical and genomic information.

*L*_1 _(lasso) and *L*_2 _(ridge) penalized estimation methods shrink the estimates of the regression coefficients towards zero relative to the maximum likelihood estimates. Both methods are similar in nature, but the results of *L*_1 _and *L*_2 _penalization can be very different. We perform the penalization only on the high-dimensional genomic covariates, the clinical covariates are handled as unpenalized mandatory variables.

The lasso shrinks the regression coefficients toward zero by penalizing the absolute values instead of their squares. The penalized log partial likelihood thus becomes $l\left(\beta ,\;\gamma \right)-\;\lambda \;\sum_{j=1}^{p}|\beta_{j}|\;.$[11].

Ridge regression [9] shrinks the regression coefficients by imposing a penalty on their squared values. The regression coefficients are estimated by maximizing the penalized log partial likelihood $l\left(\beta ,\;\gamma \right)-\lambda \sum_{j=1}^{p}\beta_{j}^{2},$where $\lambda \sum_{j=1}^{p}\beta_{j}^{2}$ is the penalty term and *l*(*β, γ*) is given by (2). Applying an *L*_2 _penalty tends to result in many small but non-zero regression coefficients, whereas penalizing with the absolute values has the effect that many regression coefficients are shrunk exactly to zero. Thus the lasso also is a variable selection method.

We applied both methods using the R package penalized [15]. In both methods the tuning parameter *λ *controls the amount of shrinkage and is obtained again by cross-validation.

### Choosing the tuning parameter

The model complexity of the prediction methods depends on a tuning parameter *λ*. We use *M*-fold cross-validation as proposed by [16] for estimating *λ*. The *M*-fold cross-validated log partial likelihood (CVPL) is given by

(6) $$\text{CVPL}\left(\lambda \right)=\overset{M}{\underset{m=1}{\sum }}\left[l\left({\hat{\beta }}_{m}\left(\lambda \right),{\hat{\gamma }}_{m}\right)-l_{m}\left({\hat{\beta }}_{m}\left(\lambda \right),{\hat{\gamma }}_{m}\right)\right],$$

where *l*(*β*, *γ*) denotes the log partial likelihood given in (2) and *l_m_*(*β*, *γ*) the log partial likelihood when the *m*th fold (*m *= 1,..., *M*) is left out.

The difference of the two terms compared in the formula is that in the right term the likelihood is evaluated without the *m*th fold, and on the left side it is evaluated with all patients. In both cases the parameters *β *and *γ *are estimated without the *m*th fold. The estimate of *β *and *γ *when the *m*th fold is left out is denoted by ${\hat{\beta }}_{m}$ and ${\hat{\gamma }}_{m}$. The optimal value of *λ *is chosen to maximize the sum of the contributions of each fold to the log partial likelihood.

### Evaluation

Next we describe how we evaluate the prediction performance of the models. We make use of three different model evaluation criteria. The whole procedure is applied to two well-known data sets. The basic idea is to split the data into a training set for model fitting and a test set for model evaluation, i.e. for determining the prediction performance. It is important to note that we have to consider several splits of the data into training and test sets due to the extreme dependence of the results on such a split (cf. [4,17]).

### Evaluation Procedure

In order to obtain a fair comparison of the prediction methods, we divide the data 100 times at random in a training and test set at the ratio of 2:1. After computing the optimal tuning parameter ${\hat{\lambda }}_{\text{train}}$ by 10-fold cross-validation using the training data, we estimate the regression coefficients ${\hat{\beta }}_{\text{train}}$ and ${\hat{\gamma }}_{\text{train}}$on the whole training data set. For each split into training data and test data, we calculate on the test set the three evaluation criteria explained in the next subsections. The results are compared with the help of boxplots and prediction error curves.

### Logrank Test

We assign patients to subgroups based on their prognosis, into one with *good *and one with *bad *prognosis. If the prognostic index $\hat{\beta }x_{i}+\hat{\gamma }z_{i}$ of patient *i *is higher, the survival time is expected to be shorter. For this reason, a patient *i *in the test set is assigned to the *high-risk *group if its prognostic index is above the median of all prognostic indices calculated on the test set. We apply a logrank test on the two prognostic groups and use the *p*-value as an evaluation criterion for the usefulness of the grouping. Boevelstad [4] points out that a disadvantage of this criterion is that it does not consider the ranking of the patients within the groups and it may not be biologically meaningful.

### Prognostic Index

The prognostic index $\hat{\beta }x_{i}+\hat{\gamma }z_{i}$ is used as a single continuous covariate in a Cox model. We fit the model $h_{i}\left(t|x_{i},\;z_{i}\right)=h_{0}\left(t\right)\exp\left(\alpha \left(\hat{\beta }x_{i}+\hat{\gamma }z_{i}\right)\right)$. Using the likelihood ratio test, we test the null hypothesis *α *= 0 versus *α *≠ 0 and assess the prediction performance with the obtained *p*-value. A small *p*-value indicates ability of the prognostic index to discriminate between short and long survivors.

### Brier Score

The prediction performance can also be assessed based on the (integrated) Brier Score that was introduced by [5] in survival context. The consistent estimate of the expected Brier Score *BS*(*t*) is defined as a function of time *t >*0 by

(7) $$\begin{array}{c} BS\left(t\right)=\frac{1}{n}\overset{n}{\underset{i=1}{\sum }}\left[\frac{Ŝ{\left(t|X_{i},Z_{i}\right)}^{2}\cdot 1\left(t_{i}\leq t\wedge \delta_{i}=1\right)}{Ĝ\left(t_{i}\right)}\right)\\ \;\;\;\;\;\;\left(+\frac{{\left(1-Ŝ\left(t|X_{i},Z_{i}\right)\right)}^{2}\cdot 1\left(t_{i}>t\right)}{Ĝ\left(t\right)}\right], \end{array}$$

where $Ŝ\left(\cdot |\;X_{i},Z_{i}\right)$ stands for the estimated survival for patient *i *and  denotes the Kaplan-Meier estimate of the censoring distribution. The estimation of $Ŝ\left(\cdot |X_{i},Z_{i}\right)$ is performed via the Breslow estimator of the cumulative baseline hazard function (see, e.g., [18], Chapter 8.8). Good predictions at time *t *are reflected by small Brier Scores. Note that the Brier Score *BS*(*t*) is dependent on the point in time *t*. The integrated Brier Score IBS, given by

(8) $$IBS=\frac{1}{t^{*}}\int_{0}^{t^{*}}BS\left(t\right)dt,$$

is a score for the average predition performance for all time points in the interval [0, *t* *]. In accordance with [6], we calculate the IBS for the two data sets for t^* ^= 10 years due to high censoring after 10 years of survival.

## Results

For investigating the relationship between microarray gene expression data and censored survival data, we analyze two published breast cancer data sets with the methods described above. In this section, we present the results for the evaluation procedure applied to these two data sets. Standard approaches focus on single genes as covariates [1,4,19]. We integrate additional biological knowledge by building models with preclustered GO groups as covariates. In order to assess the merit of this approach, we also present results for models using only genes or only GO groups as explanatory variables and combine the genomic information with the clinical data. In order to obtain a fair comparison of models with different types of genomic covariates, we only use those genes that are annotated to GO groups. We have to consider several splits of the data into training and test set due to the dependence of the results on such a split. We first present detailed results for one specific random split, then we present comprehensive results summarizing 100 random splits.

At this point we want to highlight that the proposed methods are computationally intensive. Due to the nested cross-validation procedure for obtaining the optimal tuning parameter λ and the preclustering approach, we performed all computations on the LiDOng high performance computing cluster of TU Dortmund University with 432 nodes and up to 64 GB RAM per node. The calculation takes several weeks to accumulate all results for one high dimensional data set.

### Data sets

The **Dutch breast cancer (DBC) data set **is a subset of the original data set with 24 885 gene expression measurements from *n *= 295 women with breast cancer [20]. After data pre-processing as proposed by [21] our analysis is performed with 1 890 genes, that are annotated to at least one GO group, according to the *biological process *ontology. We obtained the data from the website https://www.msbi.nl/dnn/People/Houwelingen.aspx. In total, there are 5 560 GO groups to which at least one of these genes is annotated. The mean number of genes included in these GO groups is approximately 17 genes, 90% of all GO groups contain at most 30 genes. For 79 patients an event was observed. The clinical covariates are age, size, nodes and grade.

**The Mainz cohort (MC) study **consists of *n *= 200 node-negative breast cancer patients who were treated at the Department of Obstetrics and Gynecology of the Johannes Gutenberg University Mainz between the years 1988 and 1998 [22]. All patients underwent surgery and did not receive any systemic therapy in the adjuvant setting. Gene expression profiling of the patients' RNA was performed using the Affymetrix HG-U133A array, containing 22 283 probe sets, and the GeneChip System. The normalization of the raw data was done using RMA from the Bioconductor package affy. The raw. cel files are deposited at the NCBI GEO data repository with accession number GSE11121. For covariates in the survival models, 17 834 genes and 8 587 GO groups are available. The mean number of genes included in these GO groups is approximately 102 genes, 90% of all GO groups contain at most 146 genes. There have been 47 events observed. The clinical data covers age at diagnosis, tumor size and grade as well as the estrogen receptor status.

### Exemplary analysis: One split into training and test data

We apply the model selection methods and three evaluation criteria to one specific random split of the Mainz cohort study into training and test data. Model building and evaluation are performed as explained in the evaluation procedure section. We split the 200 breast cancer patients into training set and test set, where 2/3 of the patients (in this case 133) are assigned to the training set and 1/3 (here 67) to the test set. We use the training data for estimating the tuning parameter ${\hat{\lambda }}_{\text{train}}$ and the regression coefficients ${\hat{\beta }}_{\text{train}}$train and ${\hat{\gamma }}_{\text{train}}$ and the test data for evaluation. Table 1 shows the results for the two prediction methods, using genes, GO groups, or preclustered gene groups as covariates.

**Table 1:** One random split into training and test data for the Mainz cohort study

Method	Covariates	*p*_LR_	*p*_PI_	*p*_IBS_	*λ*	sel.cov
*L*_1_	genes	0.0190	0.0017	0.1042	11.72	19
*L*_1_	GO	0.0176	0.0018	0.1103	10.75	16
*L*_1_	clustered	0.0092	0.0002	0.0830	28.53	5

*L*_2_	genes	0.0098	0.0003	0.0877	5112.08	17834
*L*_2_	GO	0.0541	0.0097	0.1022	11749.16	6530
*L*_2_	clustered	0.0690	0.0006	0.0896	96499.04	31229

Results for the two prediction methods using (i) genes, (ii) GO groups, and (iii) preclustered GO groups. For ridge regression, nearly all covariates are kept in the model since parameter estimates are unlikely to get shrunken exactly to 0.$LR\hat{=}\text{l}\text{o}\text{g}\text{r}\text{a}\text{n}\text{k}\;\text{t}\text{e}\text{s}\text{t}$, $PI\hat{=}\text{p}\text{r}\text{o}\text{g}\text{n}\text{o}\text{s}\text{t}\text{i}\text{c}\;\text{i}\text{n}\text{d}\text{e}\text{x}$,$IBS\hat{=}\text{I}\text{n}\text{t}\text{e}\text{g}\text{r}\text{a}\text{t}\text{e}\text{d}\;\text{B}\text{r}\text{i}\text{e}\text{r}\;\text{S}\text{c}\text{o}\text{r}\text{e}$,$sel.cov\hat{=}\text{n}\text{u}\text{m}\text{b}\text{e}\text{r}\;\text{o}\text{f}\;\text{s}\text{e}\text{l}\text{e}\text{c}\text{t}\text{e}\text{d}\;\text{c}\text{o}\text{v}\text{a}\text{r}\text{i}\text{a}\text{t}\text{e}\text{s}$.

This example indicates that the predictive performance of models built with GO groups alone and of models with preclustered GO groups is comparable with classical models using only genes as covariates. The *p*-values for model assessment are similar, but in addition, we have more information in the final model; annotations of preclustered GO groups can help clinicians to investigate the selected genes according to their biological function.

**Figure 1:**
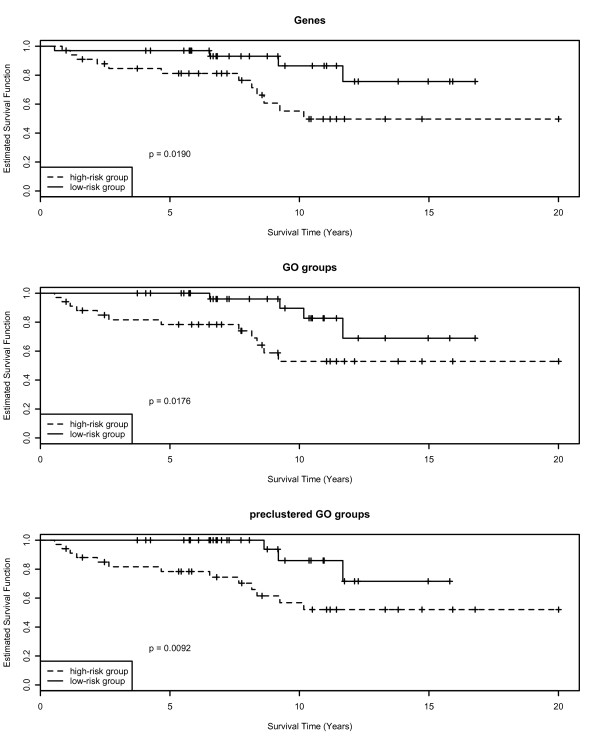
**Kaplan-Meier curves for the high-risk and low-risk groups**. Kaplan-Meier curves for the high-risk and low-risk groups defined by the estimated prognostic indices of the 67 patients in the test data set, the cutoff is defined as the median prognostic index on the test data. Genes, GO groups, and preclustered GO groups are used as covariates, respectively, and lasso regression is applied as model selection method.

For illustration of the results presented in Table 1 we show Kaplan-Meier curves for two prognostic groups of patients derived by dividing all patients according to the median prognostic index of the patients in the test set. Here we used lasso regression for model selection and the logrank test for evaluation. We compare models with genes, GO groups, and preclustered GO groups as covariates (see Figure 1).

For all three types of genomic covariates the two prognostic groups are clearly separated on the test data, with significant differences in overall survival (*p <*0.02) between the high-risk group and the low-risk group. The separation between the two groups is best when using a model containing preclustered GO groups (*p *= 0.0092).

### Comprehensive analysis: 100 splits into training and test data

We have observed high variability of the chosen tuning parameters and the parameter estimates depending on the split into training and test data. In order to quantify which covariates are consistently selected in different splits and how stable the evaluation measures are, we calculated results for 100 random splits and compared the selected genes and GO groups.

**Figure 2:**
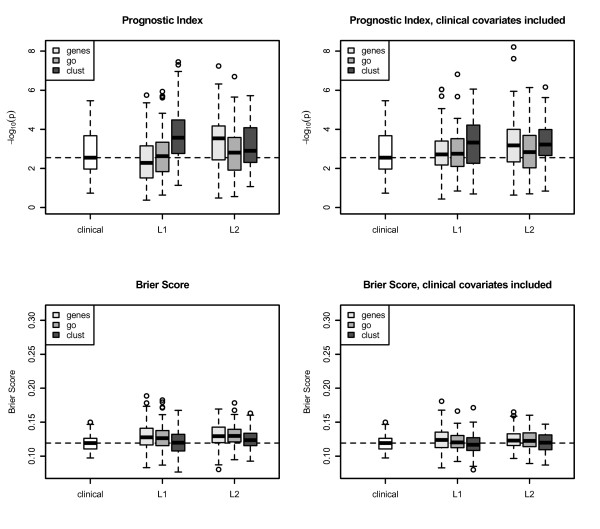
**Results: Dutch breast cancer data set**. The boxplots show results for lasso and ridge regression applied to 100 training/test splits for genes, GO groups, and preclustered GO groups for the Dutch breast cancer data set. *P*-values of the prognostic index are presented on - log_10 _scale. A small value of a criterion corresponds to a good prediction performance. The Brier Scores are calculated for 10 years follow-up. Small values of the Integrated Brier Score correspond to good prediction performance. $L_{1}\hat{=}\text{l}\text{a}\text{s}\text{s}\text{o}\;\text{r}\text{e}\text{g}\text{r}\text{e}\text{s}\text{s}\text{i}\text{o}\text{n}$,$L_{2}\hat{=}\text{r}\text{i}\text{d}\text{g}\text{e}\;\text{r}\text{e}\text{g}\text{r}\text{e}\text{s}\text{s}\text{i}\text{o}\text{n}$.

In Figures 2 (DBC) and 3 (MC), we present boxplots for the results for the two cancer data sets, after applying the evaluation procedure for lasso and ridge regression for each of the three types of genomic covariates (genes, GO groups, preclustered GO groups). Results for the clinical model are presented as a reference.

Rows of the plots correspond to two model evaluation criteria, the prognostic index and the Integrated Brier Score, and the columns correspond to two types of models: the genomic model and the genomic model with clinical covariates. Results for the logrank test are nearly the same as for the prognostic index and therefore not shown here. In each plot we show the results for the two model selection methods. The *p*-values for the prognostic index are shown on the *- *log_10 _scale, thus a value of 2, e.g., corresponds to a *p*-value of 0.01. Small values for the integrated Brier Score correspond to good prediction performance. For both evaluation criteria in all plots the horizontal line at the median indicates the reference model containing only clinical information.

**Figure 3:**
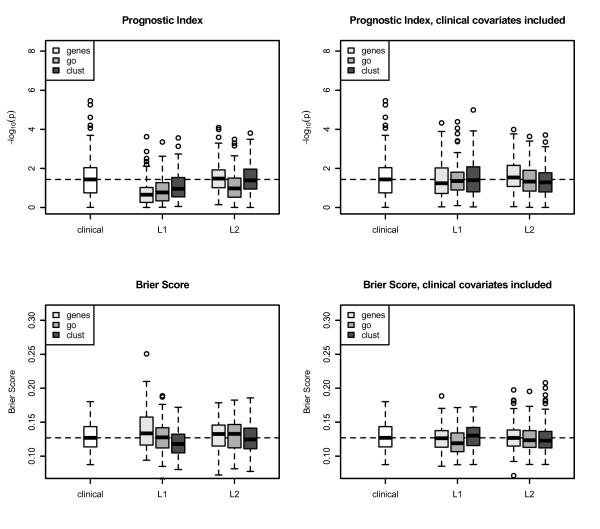
**Results: Mainz cohort study**. The boxplots show results for lasso and ridge regression applied to 100 training/test splits for genes, GO groups, and preclustered GO groups for the Mainz cohort study. *P*-values of the prognostic index are presented on *- *log_10 _scale. A small value of a criterion corresponds to a good prediction performance. The Brier Scores are calculated for 10 years follow-up. Small values of the Integrated Brier Score correspond to good prediction performance. $L_{1}\hat{=}\text{l}\text{a}\text{s}\text{s}\text{o}\;\text{r}\text{e}\text{g}\text{r}\text{e}\text{s}\text{s}\text{i}\text{o}\text{n}$,$L_{2}\hat{=}\text{r}\text{i}\text{d}\text{g}\text{e}\;\text{r}\text{e}\text{g}\text{r}\text{e}\text{s}\text{s}\text{i}\text{o}\text{n}$.

The following main statements can be deduced from the plots:

Lasso regression with preclustered GO groups has the best prediction performance for the DBC data set, see the median of the *p*-values across 100 splits in Figure 2. In the Mainz cohort study, we see the same result for the genomic model using the Brier Score for evaluation (see Figure 3).

Methods using GO groups or preclustered GO groups as covariates perform in general as well as models using only genes.

The prognostic index and the Brier Score yield similar results.

It is noticeable that for the MC study and prognostic index as performance measure the model using only genomic information is worse than the clinical model (Figure 3, upper left), but the clinical-genomic model is comparable to the clinical model.

**Figure 4:**
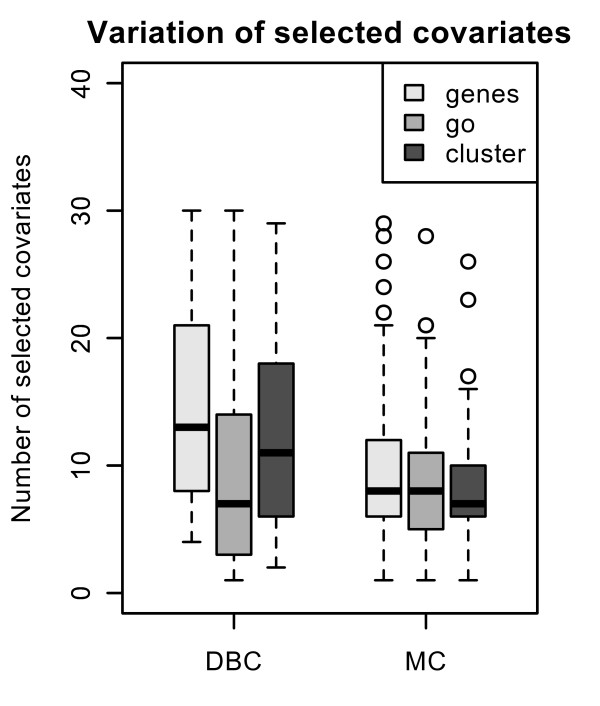
**Number of selected covariates**. Boxplots showing the number of selected covariates for lasso regression, 100 training/test splits, models with genes, GO groups and preclustered GO groups, applied to the Mainz cohort study (MC) and the Dutch breast cancer data set (DBC).

**Figure 5:**
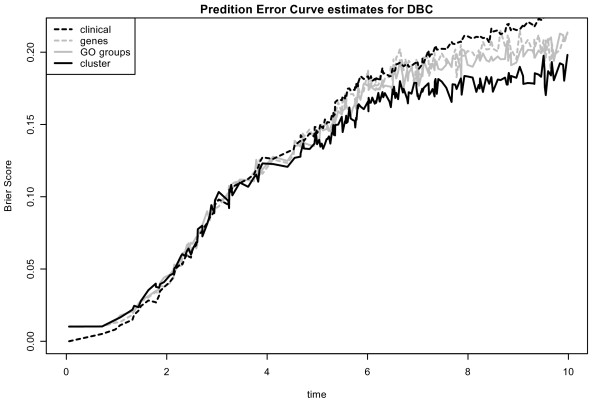
**Prediction error curves: Dutch breast cancer data set**. Prediction error curves for the DBC data set for *L*_1 _evaluation procedure. We show averaged values for the Brier Score calculated at each time point for 100 splits for models with the three different types of genomic covariates and the clinical model. A better prediction performance leads to lower curves.

**Figure 6:**
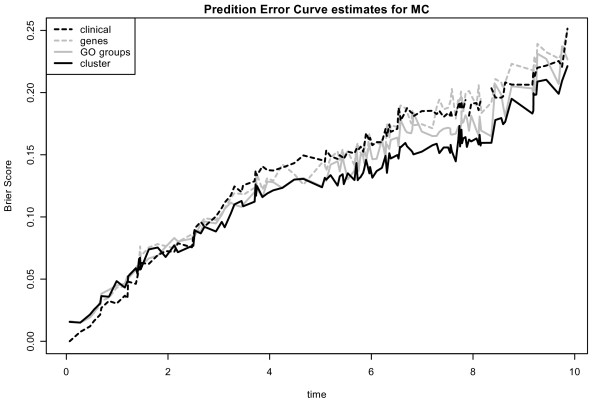
**Prediction error curves: Mainz cohort study**. Prediction error curves for the MC for *L*_1 _evaluation procedure. We show averaged values for the Brier Score calculated at each time point for 100 splits for models with the three different types of genomic covariates and the clinical model. A better prediction performance leads to lower curves.

**Figure 7:**
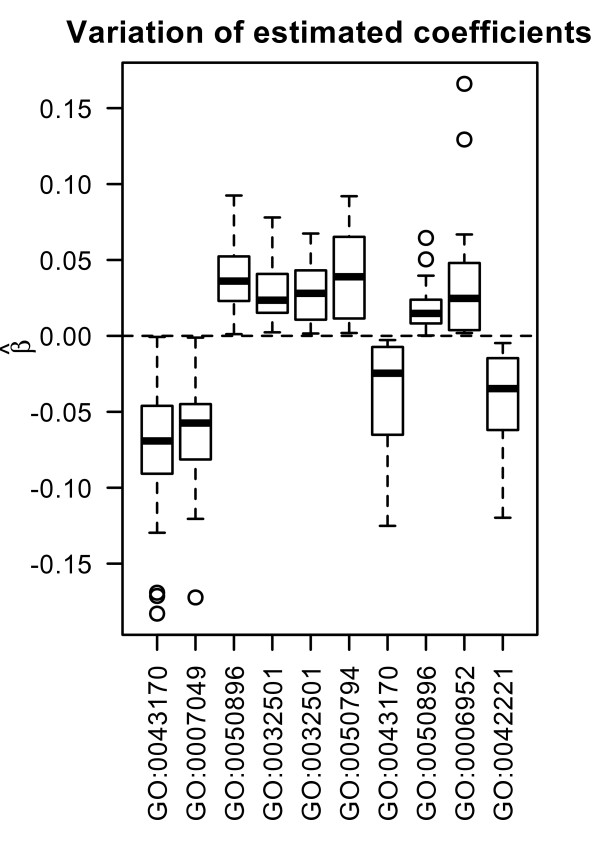
**Variation of estimated regression coefficients**. Boxplots show variation of estimated regression coefficients in the cox regression model for the most frequently chosen clusters from Table 2, represented via medoid genes.

The optimal tuning parameter varies considerably between the splits. The interquartile range for the number of chosen covariates for *L*_1 _regression and for all three different types of covariates ranges approximately from 5 to 12 for the Mainz cohort study and from 3 and 20 for the DBC data set (see Figure 4). There is a higher variance on the number of chosen covariates for the DBC data set. Next, we have a closer look at the run of the curves of the Brier Score over time for *L*_1 _models with preclustered GO groups in comparison to the other models. Prediction error curves [5,23] (averaged values for the Brier Score calculated at each time point for 100 splits) for models with the three different types of genomic covariates are shown in Figure 5 and 6 for the DBC data set and the MC study, respectively. The performance of the clinical model serves as reference. For both data sets, the model with preclustered GO groups has in comparison with the clinical model a better prediction performance over time. The preclustered models outperform the clinical models, starting at four years for the DBC data set and at three years for the MC study. The other two genomic models are also inferior to the preclustered models. Furthermore, we investigate which preclustered groups are most frequently selected across all 100 splits. Table 2 contains the numbers of the most frequently selected covariates, the corresponding GO groups with GO IDs [7] and further information concerning the medoid gene, the cluster size and the annotation for the GO groups that are helpful for the biologist. We observe that most of the chosen cluster are subgroups of large GO groups and consist of more 100 genes. The value of the *effect *indicates whether a high value of the corresponding covariate has an increasing (+1) or decreasing (-1) influence on patients' risk to die. For a detailed analysis of the effects the boxplots in Figure 7 show the variation of the estimated regression coefficients in the cox regression model for the most frequently chosen clusters, represented via medoid genes. First of all, the direction of the effect among all splits into training and test data is stable. From this it follows that a detected cluster has a consistent effect on patients' survival - either positive or negative. The first two clusters (from GO:0043170 and GO:0007049) shown in Table 2 are chosen in more than 80 percent of the splits into training and test data. Their parameter estimates are negative, i.e. high expression values of the included genes lead to reduced risk to die and thus to longer survival.

**Table 2:** Top 10 selected covariates for preclustered GO-groups according to 100 splits into training and test data

count	GO	effect	medoid	clustersize	annotation
85	GO:0043170	-1	209258_s_at	410	macromolecule metabolic process
81	GO:0007049	-1	210052_s_at	222	cell cycle
74	GO:0050896	+1	211908_x_at	102	response to stimulus
52	GO:0032501	+1	212195_at	310	multicellular organismal process
40	GO:0032501	+1	210935_s_at	362	multicellular organismal process
21	GO:0050794	+1	210417_s_at	312	regulation of cellular process
18	GO:0043170	-1	211693_at	434	macromolecule metabolic process
18	GO:0050896	+1	204118_at	230	response to stimulus
16	GO:0006952	+1	203535_at	27	defense response
15	GO:0042221	-1	219140_s_at	39	response to chemical stimulus

Names are GenBank IDs for the medoid genes and GO IDs for GO groups [7]. The first column corresponds to the selected number for the covariate across 100 splits into training and test data for *L*1 regression on the Mainz cohort study. The value of the *effect *indicates whether the covariate has an increasing(+1) or decreasing (*-*1) effect on patients' risk to die.

## Discussion

The typical challenge when relating survival times to gene expression measurements is a relatively small number of individuals compared to a large number of predictors. In this case the use of classical approaches is not possible. In accordance with [4], the lasso regression method seems most suitable and promising: its prediction performance is slightly better compared to ridge regression and the solution is sparse [4] and [12]. show that ridge regression performs better than all the other methods. In our analysis, ridge regression leads in general to comparable but not better results compared to the lasso. However, an important disadvantage of this method is that it does not select variables. We observe relevant differences between high-risk and low-risk patients, but there are too many genes or GO groups to be further investigated. The preclustering approach is beneficial concerning prediction performance in the lasso setting and leads to comparable results in the other models. However, a main benefit of preclustering is that we detect genes with similar expression patterns and that these gene subgroups are correlated with survival. In addition, we can have a detailed view on the GO groups containing the preclustered subgroups. Table 2 shows that the cluster sizes as well as the corresponding GO groups are quite large. However, in this case the selection of the top 4 clusters is quite stable. For gaining further biological insight a more detailed analysis of the composition of these clusters is required and promising.

In terms of the Brier Score, we showed that the prediction performance of models using clinical, genomic or both information is comparable. It seems that these different kind of covariates contain an overlap of information for predicting survival.

## Conclusions

Our comparative study shows that different model selection procedures can be used to identify genes and (preclustered) GO groups related to survival outcomes and to build models for predicting survival times of future patients.

The integration of GO groups is useful, since they contain aggregated information of biological function and thus are often more informative than single genes. It is encouraging that in terms of prediction performance, our results obtained with (preclustered) GO groups as predictors are comparable to those using only genes as predictors. Thus the potentially improved interpretability makes these models with GO groups competitive. We demonstrated that this result holds true also for models using GO groups and not only genes. Our agenda in the present work was:

Constructing models with a relatively small subset of relevant covariates that are enriched with additional gene group information in terms of the Gene Ontology.

Presenting a new approach of preclustering genes from one functional group due to different expression profiles within one GO group.

Comparing prediction rules for the three types of covariates (genes, gene groups, preclustered gene groups).

Adding clinical information and comparing the results to single use of genomic data.

The next step for improving our models is to integrate more detailed information concerning the hierarchically structured gene ontology. For coping with high correlations between GO groups one can follow the approach of [24] where correlations between neighboring GO groups in the GO graph are iteratively removed. Finally, in future projects, the biological interpretation of the identified gene groups will include not only the interpretation of the (preclustered) GO groups according to overall function, but it is also helpful to take a closer look at the single genes contained in these gene groups.

## Availability

We make use of the R package penalized [15] that provides algorithms for penalized estimation in Cox proportional hazards models. The package is freely available from http://cran.r-project.org[25]. R code for model selection and evaluation is available at http://www.statistik.tu-dortmund.de/survivalGO.html.

## Authors' contributions

KK and JR developed the ideas for the manuscript, KK and ML performed the statistical and computational analyses. MS and JGH generated and provided the Mainz cohort data, all authors read and approved the manuscript.

## References

[B1] Gui J Li H Penalized Cox regression analysis in the high-dimensional and low-sample size settings, with applications to microarray gene expression data Bioinformatics 2005 21 13 3001 3008 10.1093/bioinformatics/bti422 15814556

[B2] Boulesteix AL Porzelius C Daumer M Microarray-based classification and clinical predictors: on combined classifiers and additional predictive value Bioinformatics 2008 24 15 1698 1706 10.1093/bioinformatics/btn262 18544547

[B3] Binder H Schumacher M Allowing for mandatory covariates in boosting estimation of sparse high-dimensional survival models BMC Bioinformatics 2008 9 14 10 19 18186927 10.1186/1471-2105-9-14 PMC2245904

[B4] Bøvelstad HM Nygård S Størvold HL Aldrin M Frigessi A Lingjaerde OC Predicting survival from microarray data-a comparative study Bioinformatics 2007 23 16 2080 2087 10.1093/bioinformatics/btm305 17553857

[B5] Graf E Schmoor C Sauerbrei W Schumacher M Assessment and comparison of prognostic classification schemes for survival data Stat Med 1999 18 17-18 2529 2545 10.1002/(SICI)1097-0258(19990915/30)18:17/18<2529::AID-SIM274>3.0.CO;2-5 10474158

[B6] Schumacher M Binder H Gerds T Assessment of survival prediction models based on microarray data Bioinformatics 2007 23 14 1768 1774 10.1093/bioinformatics/btm232 17485430

[B7] Gene Ontology Consortium The Gene Ontology project in 2008 Nucleic Acids Res 2008 36 Database D440 D444 17984083 10.1093/nar/gkm883 PMC2238979

[B8] Cox DR Regression models and life tables (with discussion) J R Stat Soc B 1972 34 2 187 220

[B9] Hoerl AE Kennard RW Ridge regression: biased estimation of nonorthogonal problems Technometrics 1970 12 55 67 10.2307/1267351

[B10] Tibshirani R Regression shrinkage and selection via the lasso J R Stat Soc B 1996 58 267 288

[B11] Tibshirani R The lasso method for variable selection in the Cox model Stat Med 1997 16 4 385 395 10.1002/(SICI)1097-0258(19970228)16:4<385::AID-SIM380>3.0.CO;2-3 9044528

[B12] Bøvelstad HM Nygård S Borgan O Survival prediction from clinico-genomic models-a comparative study BMC Bioinformatics 2009 10 413 10.1186/1471-2105-10-413 20003386 PMC2811121

[B13] Kaufman L Rousseeuw PJ Finding Groups in Data - An introduction to cluster analysis 1995 Wiley, New York

[B14] Haan JRD Piek E van Schaik RC de Vlieg J Bauerschmidt S Buydens LMC Wehrens R Integrating gene expression and GO classification for PCA by preclustering BMC Bioinformatics 2010 11 158 10.1186/1471-2105-11-158 20346140 PMC2860362

[B15] Goeman Jpenalized: L1 (lasso) and L2 (ridge) penalized estimation in GLMs and in the Cox model 2008 [R package version 0.9-23]

[B16] Verweij PJ van Houwelingen HC Cross-validation in survival analysis Stat Med 1993 12 24 2305 2314 10.1002/sim.4780122407 8134734

[B17] Ein-Dor L Zuk O Domany E Thousands of samples are needed to generate a robust gene list for predicting outcome in cancer Proc Natl Acad Sci USA 2006 103 15 5923 5928 10.1073/pnas.0601231103 16585533 PMC1458674

[B18] Klein JP Moeschberger ML Survival Analysis Techniques for Censored and Truncated Data 2003 Second

[B19] Haibe-Kains B Desmedt C Sotiriou C Bontempi G A comparative study of survival models for breast cancer prognostication based on microarray data: does a single gene beat them all? Bioinformatics 2008 24 19 2200 2208 10.1093/bioinformatics/btn374 18635567 PMC2553442

[B20] van de Vijver MJ He YD van't Veer LJ Dai H Hart AAM Voskuil DW Schreiber GJ Peterse JL Roberts C Marton MJ Parrish M Atsma D Witteveen A Glas A Delahaye L van der Velde T Bartelink H Rodenhuis S Rutgers ET Friend SH Bernards R A gene-expression signature as a predictor of survival in breast cancer N Engl J Med 2002 347 25 1999 2009 10.1056/NEJMoa021967 12490681

[B21] van Houwelingen HC Bruinsma T Hart AAM Veer LJV Wessels LFA Cross-validated Cox regression on microarray gene expression data Stat Med 2006 25 18 3201 3216 10.1002/sim.2353 16143967

[B22] Schmidt M Hasenclever D Schaeffer M Boehm D Cotarelo C Steiner E Lebrecht A Siggelkow W Weikel W Schiffer-Petry I Gebhard S Pilch H Gehrmann M Lehr HA Koelbl H Hengstler JG Schuler M Prognostic effect of epithelial cell adhesion molecule overexpression in untreated node-negative breast cancer Clin Cancer Res 2008 14 18 5849 5855 10.1158/1078-0432.CCR-08-0669 18794096

[B23] Gerds TA Schumacher M Consistent estimation of the expected Brier score in general survival models with right-censored event times Biom J 2006 48 6 1029 1040 10.1002/bimj.200610301 17240660

[B24] Alexa A Rahnenführer J Lengauer T Improved scoring of functional groups from gene expression data by decorrelating GO graph structure Bioinformatics 2006 22 13 1600 1607 10.1093/bioinformatics/btl140 16606683

[B25] R Development Core Team R: A Language and Environment for Statistical Computing 2010 R Foundation for Statistical Computing, Vienna, Austria

